# Diagnostic Value of Fluorescence Methods, Visual Inspection and Photographic Visual Examination in Initial Caries Lesion: A Systematic Review and Meta-Analysis

**DOI:** 10.3390/dj9030030

**Published:** 2021-03-06

**Authors:** Mai Thi Giang Thanh, Ngo Van Toan, Do Thi Thanh Toan, Nguyen Phu Thang, Ngoc Quang Dong, Nguyen Tien Dung, Phung Thi Thu Hang, Le Quynh Anh, Nguyen Thu Tra, Vo Truong Nhu Ngoc

**Affiliations:** 1 Department of Clinical science, Hadong Medical College, Hanoi 100000, Vietnam; maithigiangthanh@gmail.com; 2 School of Odonto Stomatology, Hanoi Medical University, Hanoi 100000, Vietnam; phuthang@hmu.edu.vn (N.P.T.); dr.domnguyen@gmail.com (N.T.D.); phunghang3001@gmail.com (P.T.T.H.); qule7436@uni.sydney.edu.au (L.Q.A.); 3 Institute for Preventive Medicine and Public Health, Hanoi Medical University, Hanoi 100000, Vietnam; ngovantoan@hmu.edu.vn (N.V.T.); dothithanhtoan@hmu.edu.vn (D.T.T.T.); 4 Hanoi Medical University Hospital, Hanoi Medical University, Hanoi 100000, Vietnam; 5 Department of Oral and Maxillofacial Surgery, Shimane University Faculty of Medicine, Shimane 693-8501, Japan; dongngocquang1987@gmail.com; 6 Department of Plastic, Reconstructive and Cosmetic Surgery, National Hospital of Odonto-Stomatology, Hanoi 100000, Vietnam; 7 School of Dentistry, Faculty of Medicine and Health, The University of Sydney, New South Wales 2006, Australia

**Keywords:** fluorescence, initial tooth caries, photographic visual examination, visual inspection

## Abstract

This systematic review and meta-analysis aimed to investigate the efficacy of fluorescence-based methods, visual inspections, and photographic visual examinations in initial caries detection. A literature search was undertaken in the PubMed and Cochrane databases. Preferred Reporting Items for Systematic Review and Meta-Analyses (PRISMA) guidelines were followed, and eligible articles published from 1 January 2009 to 30 October 2019 were included if they met the following criteria: they (1) assessed the accuracy of methods of detecting initial tooth caries lesions on occlusal, proximal, or smooth surfaces in both primary and permanent teeth (in clinical); (2) used a reference standard; (3) reported data regarding the sample size, prevalence of initial tooth caries, and accuracy of the methods. Data collection and extraction, quality assessment, and data analysis were conducted according to Cochrane standards Quality Assessment of Diagnostic Accuracy Studies-2. Statistical analyses were performed using Review Manager 5.3 and STATA 14.0. A total of 12 eligible articles were included in the meta-analysis. The results showed that the sensitivity and specificity of fluorescence-based methods were 80% and 80%, respectively; visual inspection was measured at 80% and 75%, respectively; photographic visual examination was measured at 67% and 79%, respectively. We found that the visual method and the fluorescence method were reliable for laboratory use to detect early-stage caries with equivalent accuracy.

## 1. Introduction

Noncavitated lesions, referring to initial caries lesion development, are characterized by alterations in color, glossiness, or surface structure—resulting from demineralization before the appearance of visible breakdown in the tooth surface [1].

According to Makhija in 2014 [2], 96% of early tooth lesions could be effectively treated by noninvasive interventions. In keeping with the trend of modern dentistry toward minimal intervention, early diagnosis of dental caries is considered a top priority goal. Diagnosis of dental caries is based mainly on symptoms and clinical signs; however, diagnosis is challenging and results may vary, depending on the presentation of the disease [3]. Detecting early lesions and monitoring signs of progression can be problematic, even for experienced dentists [4,5]. Therefore, the selection of a feasible, easy-to-apply and highly reliable method for early dental caries diagnosis is essential. The limitations of radiographs and clinical visual or tactile examination are that they are unable to detect whether the minimal enamel change is characteristic of early caries progression or remineralization [6]. Moreover, radiographs show low sensitivity for dentin caries (0–2 versus 3) [7]. According to Abogazalah N [8], the systematic review and meta-analysis of the visual inspection [9] and radiographic methods [10] showed diagnostic efficiency in detection of tooth decay on the proximal surface of the teeth, with low sensitivity and high specificity. Along with advances in science and technology, several new methods can assist the diagnosis of early-stage tooth decay, e.g., laser fluorescence [4] and optical coherence tomography [4,7]. Although these new methods offer various choices for early detection of tooth decay in modern dentistry, reports regarding their effectiveness have delivered contradictory results [8,9,10]. Consequently, it is very difficult to choose the best method for clinical application.

Fluorescence-based methods for caries detection are in common use because they are capable of quantifying the mineral loss of hard dental tissues. The mechanisms of the DIAGNOdent 2095 (LF, KaVo, Biberach, Germany) and DIAGNOdent 2190 pens (LFpen, KaVo) are based primarily on fluorescence absorption of products released by bacteria in carious surfaces, which are illuminated by a diode laser with a 655 nm wavelength [11]. An intraoral fluorescence camera (FC, VistaProof) produces blue light at 405 nm to capture and digitalize images from the teeth while they are emitting fluorescence [12]. In the initial stage of carious lesions, red porphyrin fluorescence is emitted. This fluorescence is not emitted by intact enamel [13]. The quantitative light-induced fluorescence method (QLF) uses light with wavelengths of 405 nm to stimulate yellow fluorescence at wavelengths above 520 nm. Its diagnostic capability is based on the intensity of natural fluorescence of a tooth which is decreased by scattering due to caries lesions [14].

In daily clinical practice, dental caries are usually detected by visual inspection [15], a method which is simple and cost-effective. Nevertheless, visual examination has some disadvantages, mainly associated with its subjective nature [16]; i.e., the inconsistent interpretation of clinical characteristics of carious lesions [15]. A meta-analysis by Gimenez, T [9] showed that visual detection of carious lesions has been well-studied, and has been suggested as an exclusive method in clinical practice [9] for its high overall accuracy. However, there has been no meta-analysis regarding the diagnosis of initial lesion caries by visual examination.

Dental photography is a very technique-sensitive method, owing to distance, the humid, dark environment of the mouth, and the interaction between light and dental tissues. Some authors have published articles reporting the diagnosis of early stages caries by photograph, but there is no consensus about the effectiveness of this method.

Therefore, this systematic review and meta-analysis were purposed to investigate the overall diagnostic accuracy of image-based, fluorescence-based, and visual inspection-based detection methods in early dental caries in primary and permanent teeth. We also investigated possible sources of publication bias.

## 2. Materials and Methods

The data searching strategy was based on PRISMA’s guideline (Preferred Reporting Items for Systematic Review and Meta-Analyses [15]) in order to minimize the number of missing articles and increase the clarity and transparency of the systematic review [17]. The research questions were constructed based on PICOS [18]: (P) participants: early dental caries; (I) intervention: image-based detection, fluorescence methods, and visual inspection in early dental caries; (C) comparison or control group: gold standard; (O) outcome: accuracy, sensitivity, specificity, area under the receiver operating characteristic (ROC); (S) study design: caries lesions on occlusal, approximal or smooth surfaces, in both primary or permanent human teeth, in the clinical setting.

### 2.1. Search Strategy

The process of building the database was carried out on Cochrane library and PubMed from 1 January 2009 to 30 October 2019 using the terms (which were divided into 3 groups): sensitivity and specificity; early dental caries; methods of detecting early dental caries. Boolean operators, such as “AND” and “OR”, were used appropriately in each group. Post-research studies were input to Endnote X9 produced by Clarivate Analytics software to store, organize and manage publication information. The search terms used for each database are listed in Appendix A (Table A1) and Appendix B (Table A2).

### 2.2. Selection Criteria

Articles were excluded if they met these criteria: irrelevance; review or conceptual articles; non-human teeth; not related to early dental caries; not related to diagnosis; radicular caries lesions; secondary caries lesions; artificial lesions; methodology articles; method not based on laser fluorescence (for LF only) or method not based on photograph/smart phone or method not based on oral/clinical examination (for clinical examination only; without validation; not about performance).

We also excluded studies that were not in English or unavailable for full text articles. 

Studies were screened by two independent researchers. Disagreements were resolved by discussion and expert consultation.

### 2.3. Data Extraction and Quality Assessment

Extracted data included first author’s name, publication year, sample size and outcome data (sensitivity and specificity), the values of true positives (TP), true negatives (TN), false positives (FP) and false negatives (FN) if reported.

Two researchers independently assessed the quality of the study using Quality Assessment of Diagnostic Accuracy Studies-2 (QUADAS-2) [19] based on four domains (“Patient Selection”, “Index Test”, “Reference Standard”, and “Flow and Timing”) in two categories (“Risk of Bias” and “Applicability Concerns”).

### 2.4. Statistical Analyses

Statistical analyses were performed using Review Manager Software (RevMan Version 5.3, the Nordic Cochrane Centre, the Cochrane Collaboration, Copenhagen, Denmark) and STATA 14.0 software. Review Manager 5.3 software was employed to assess the quality of enrolled studies.

## 3. Results

### 3.1. Study Selection

**Figure 1:**
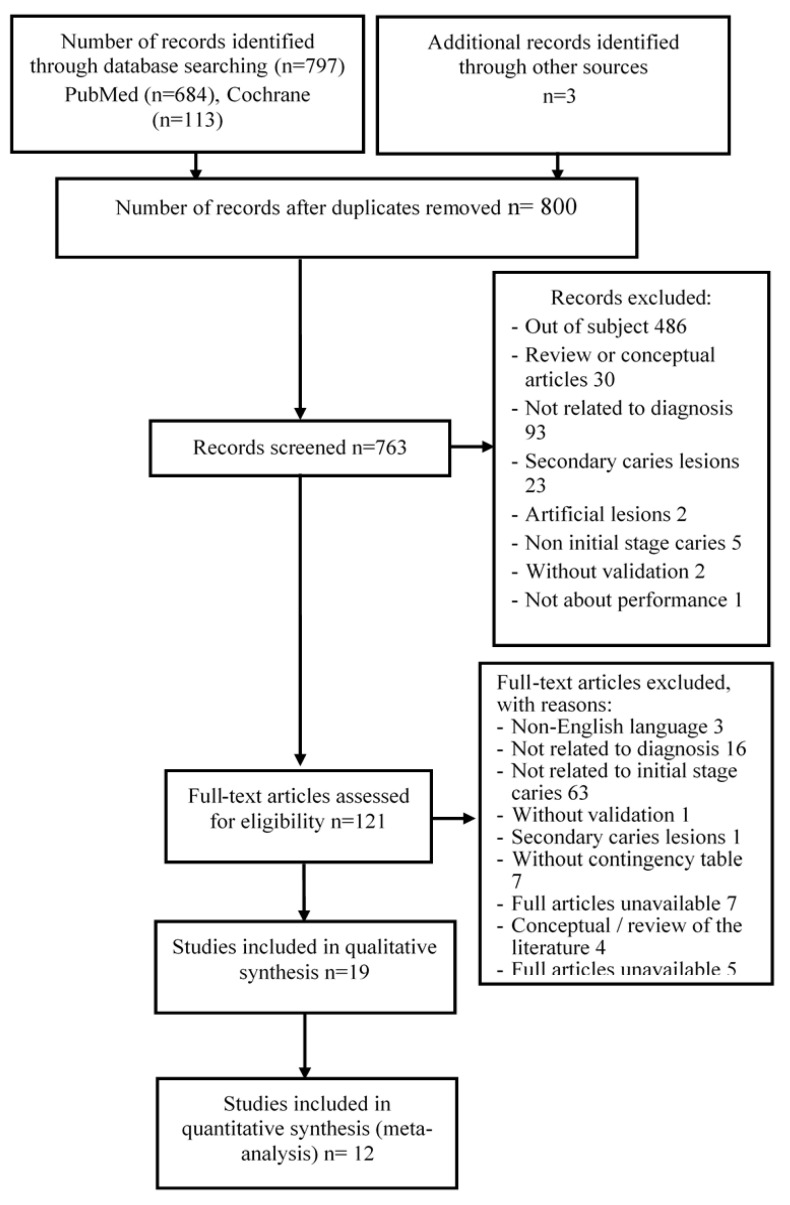
Flow chart of study selection.

Among 797 records, 31 were eliminated due to duplication. After screening titles and abstracts, 642 were excluded and further 109 articles were removed due to specific reasons shown in a flow diagram (Figure 1). This has led to our finalized database of 12 studies for quantitave analysis.

### 3.2. Study Characteristics

Publication year ranged from 2011 to 2018. Most of the articles used visual inspection or histological examination in occlusal surface as reference standard. Out of the twelve selected articles, three articles used visual photographic examination, ten articles used fluorescence methods, and five articles utilized visual inspection in initial caries detection. A summary containing characteristics of each included study was provided in Appendix C (Table A3).

### 3.3. Study Quality Assessment

Using QUADAS-2, 75% of studies had a high risk of bias in the patient selection domain and 50% of studies had a high risk of bias in the index test domain. In the reference standard domain, 58% of studies had a high risk of bias while 17% of studies did not present enough information. Of the studies, 8% had a high risk of bias in the flow and timing domain. All studies had an appropriate sample, reference standard and index test with the review question (Table 1).

### 3.4. Sensitivity and Specificity

**Figure A1:**
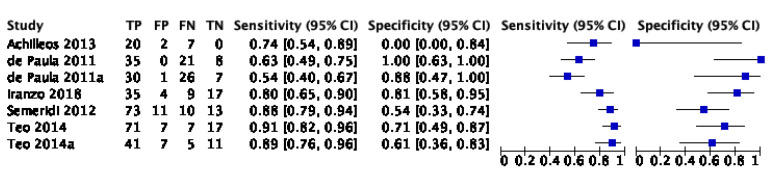
Visual inspection.

**Figure A2:**
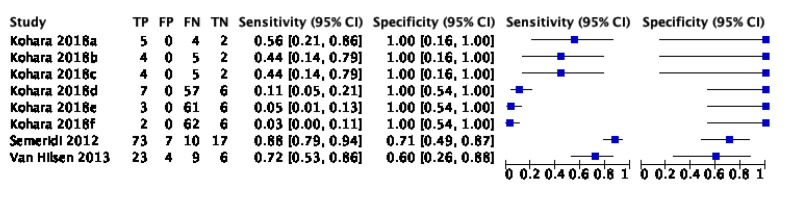
Photographic visual examination.

**Figure A3:**
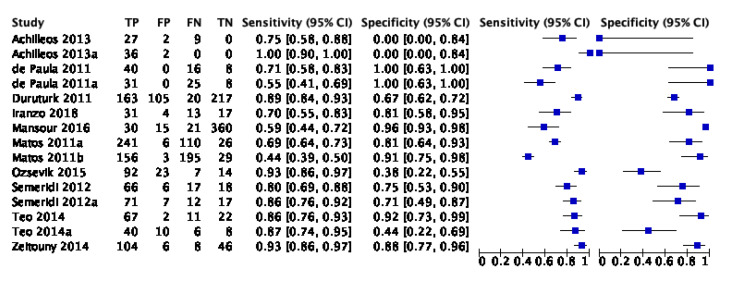
Fluorescence-based methods.

The sensitivity and specificity of each study included in the review were presented in Appendix D (Figure A1, Figure A2 and Figure A3). The overall sensitivity and specificity of visual inspection were 0.8 (95% CI: 0.69–0.88) and 0.75 (95% CI: 0.58–0.86), respectively. The overall sensitivity and specificity of visual photographic examination were 0.67 (95% CI: 0.45–0.82) and 0.79 (95% CI: 0.5–0.93), respectively. The sensitivity and specificity of fluorescence methods were 0.8 (95% CI: 0.71–0.87) and 0.8 (95% CI: 0.68–0.88), respectively.

### 3.5. Summary Receiver Operating Characteristic (sROC) Curves

**Figure 2:**
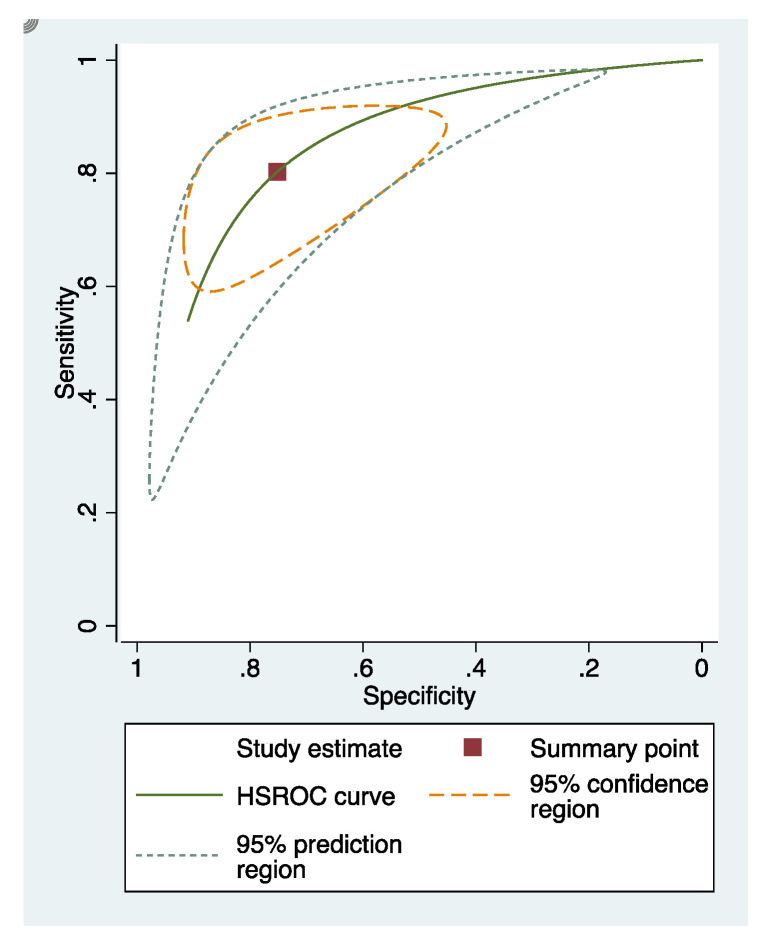
sRoc curves of visual inspection.

**Figure 3:**
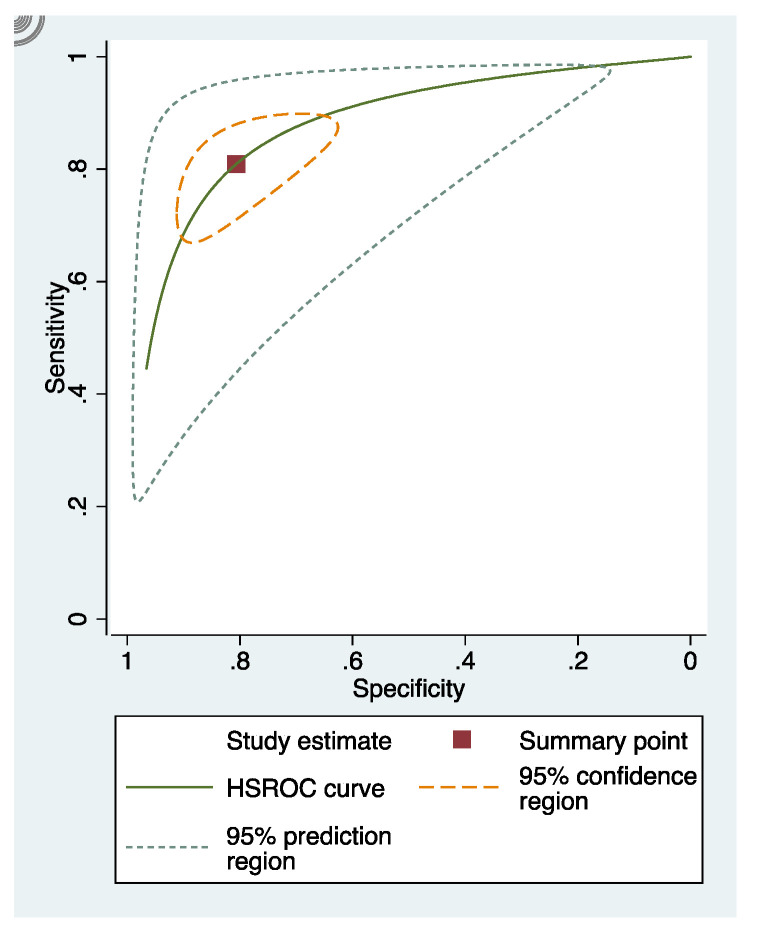
sRoc curves of fluorescence-based methods.

**Figure 4:**
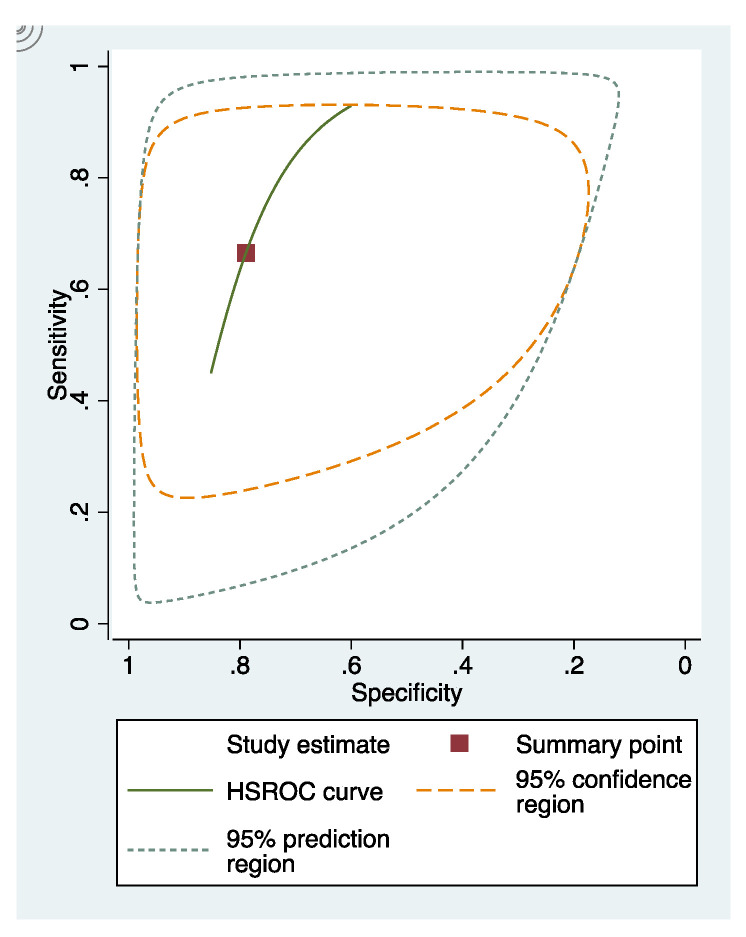
sRoc curves of photographic visual examination.

In these groups, analysis area under the curve (AUC) provided more information about the research results (Figure 2, Figure 3 and Figure 4).

**Figure 5:**
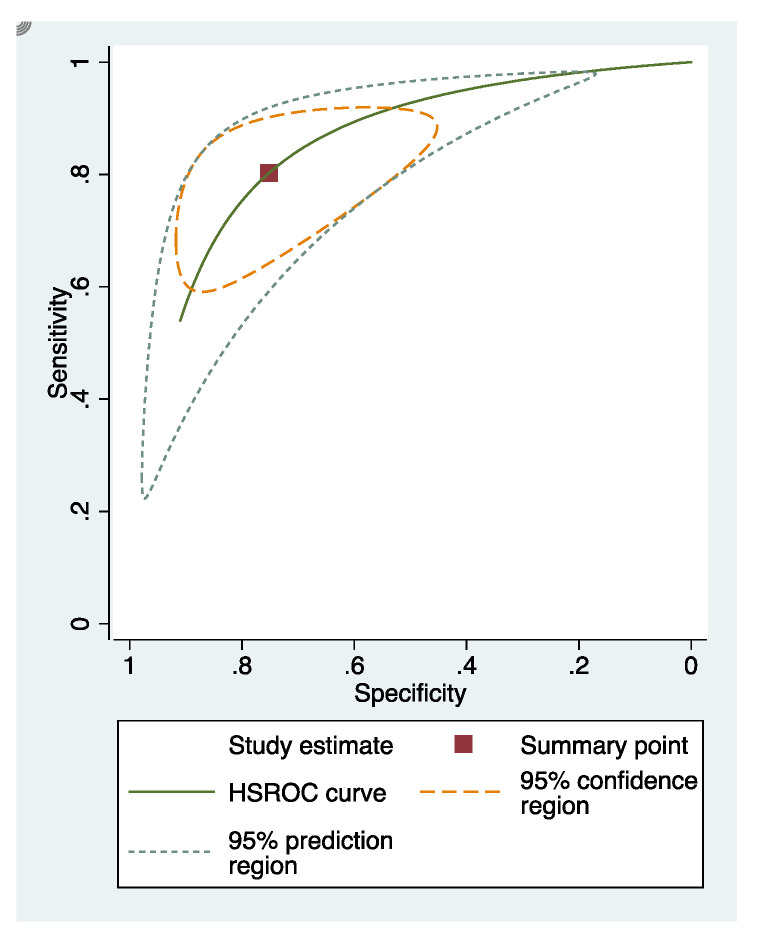
sRoc curves of visual inspection on occlusal surfaces.

**Figure 6:**
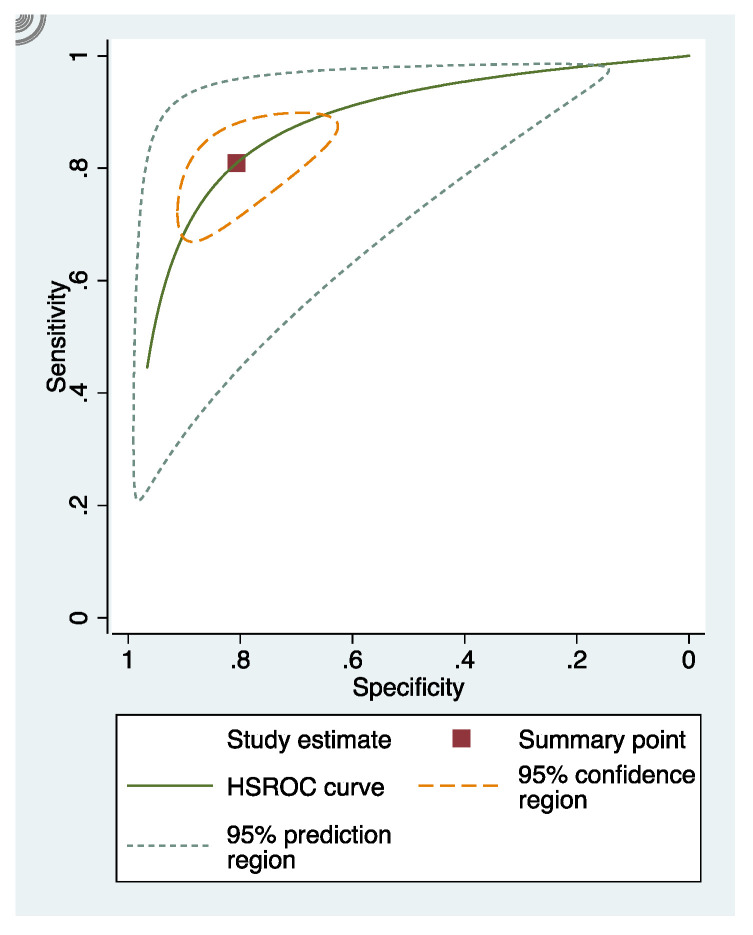
sRoc curves of fluorescence-based methods on occlusal surfaces.

**Figure 7:**
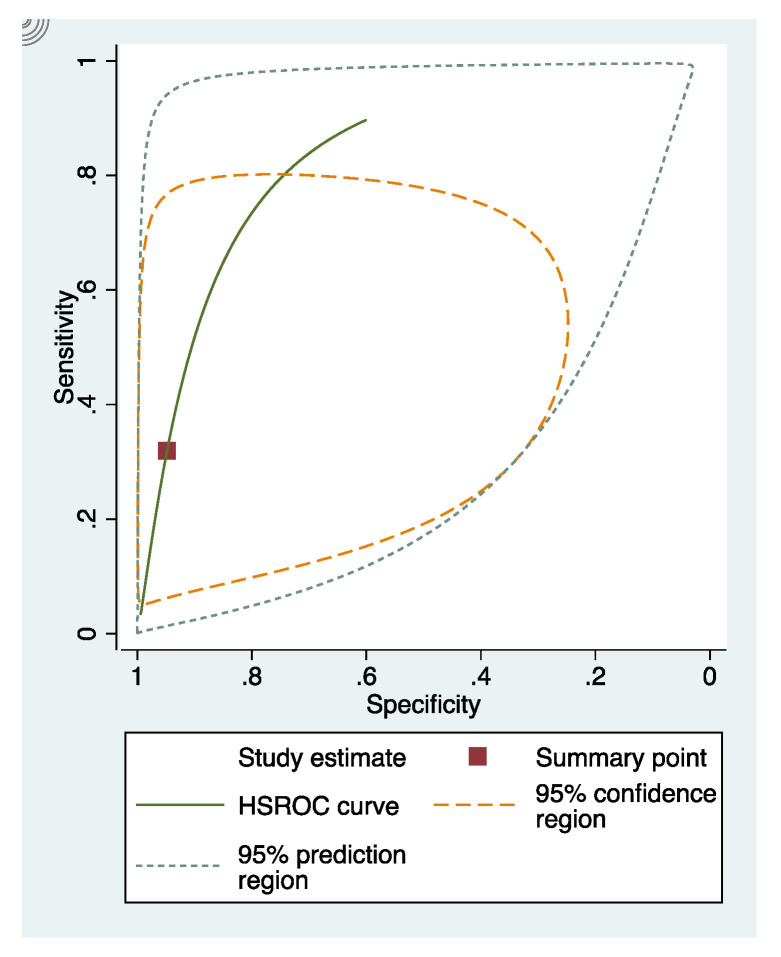
sRoc curves of photographic visual examination on occlusal surfaces.

sRoc curves of visual inspection, fluorescence-based methods, photographic visual examination on occlusal surfaces of teeth are illustrated in Figure 5, Figure 6 and Figure 7. The pooled sensitivities and specificities of visual inspection and fluorescence methods were higher than that of photographic visual examination. Regarding the occlusal surface of teeth, the values of I-squared were high at all methods (72% to 98%). The sensitivity and specificity of visual inspection were 0.8 (95% confident interval: 0.72–0.88) and 0.75 (95% CI: 0.64–0.87), respectively. The sensitivity and specificity of fluorescence methods were 0.8 (95% CI: 0.74–0.87) and 0.8 (95% CI: 0.72–0.89), respectively. The sensitivity and specificity of visual photographic examination were 0.3 (95% CI: 0.1–0.56) and 0.9 (95% CI: 0.85–0.99), respectively.

**Figure 8:**
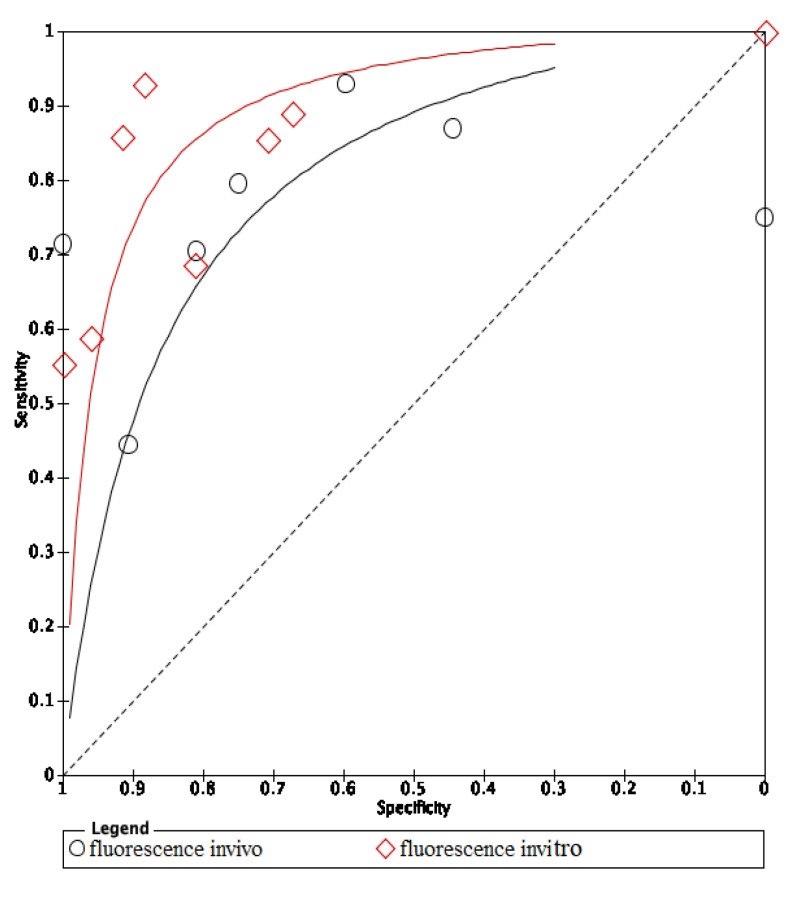
Comparison between in vitro and in vivo of fluorescence methods.

**Figure 9:**
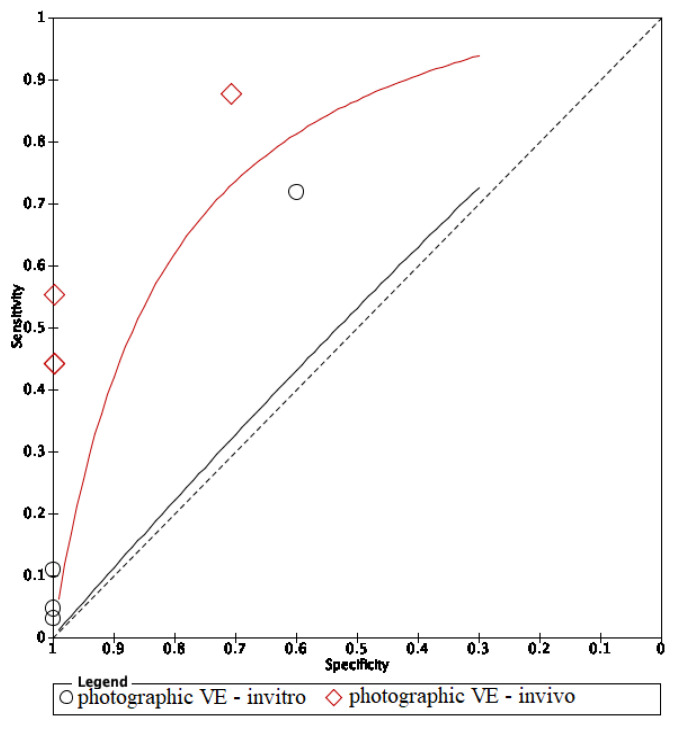
Comparison between in vitro and in vivo of visual photographic examination methods.

The area under the curve (AUC) of in vivo fluorescence was higher in in vitro fluorescence. On the other hand, methods using in vivo visual photographic examination had lower AUC of in vitro visual photographic examination (Figure 8 and Figure 9).

**Figure 10:**
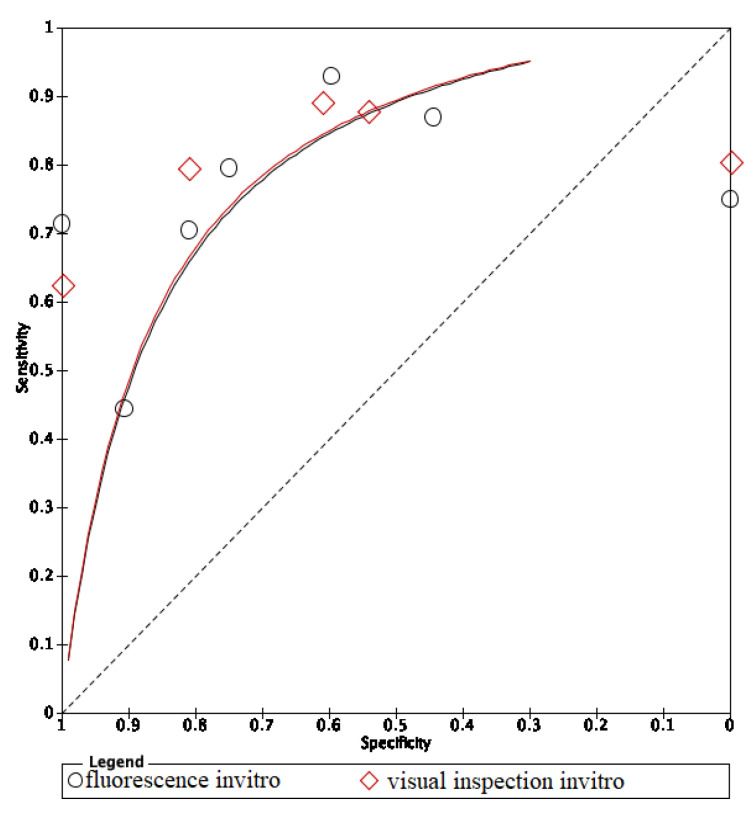
In vitro comparison of fluorescence and visual inspection.

**Figure 11:**
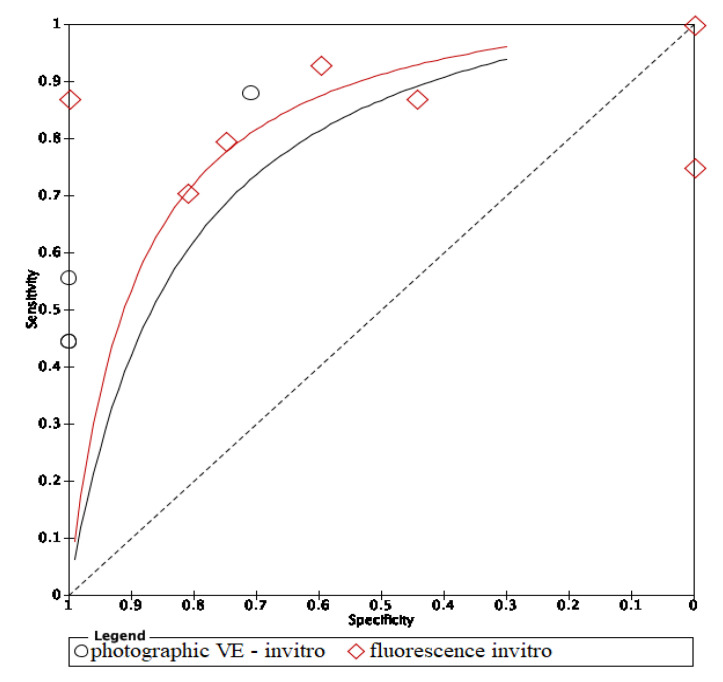
In vitro comparison of photographic visual examination and fluorescence.

**Figure 12:**
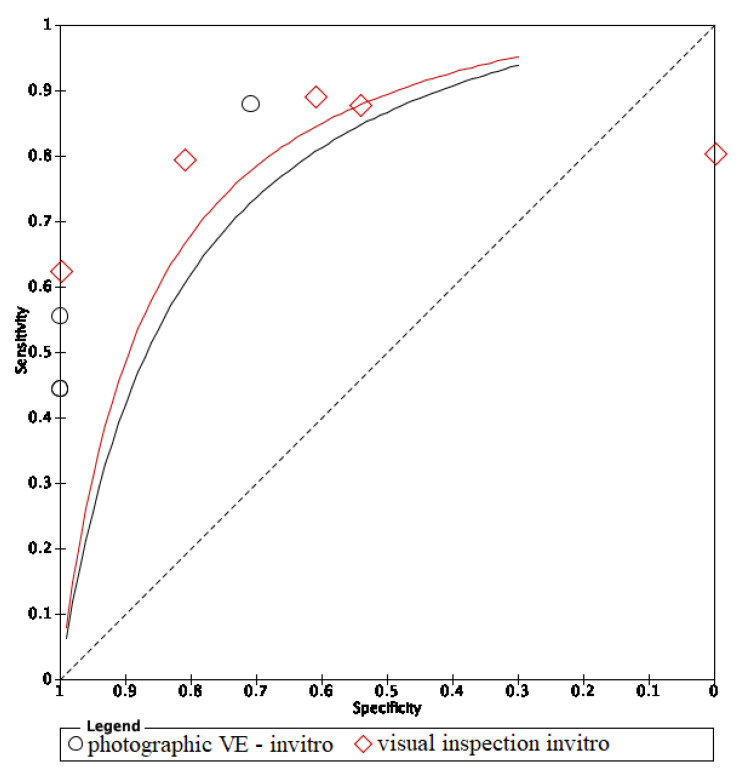
In vitro comparison of visual inspection and photographic visual examination.

The ROC curves of in vitro visual inspection and fluorescence coincided displaying the equivalence in both methods’ accuracy (Figure 10, Figure 11 and Figure 12). The AUC of in vitro visual photographic examination was lower than that of in vitro fluorescence and visual detection.

**Figure 13:**
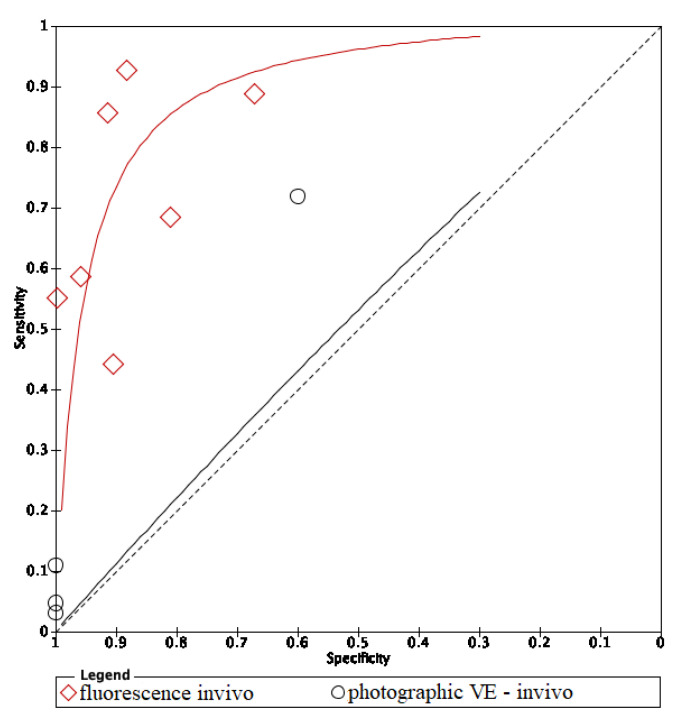
In vivo comparison of fluorescence and image methods.

The ROC curve of in vivo fluorescence is directed towards the upper left corner than the curve of in vivo visual photographic examination (Figure 13).

## 4. Discussion

Concerning advanced adjunct methods to detect dental decay, two previous systematic reviews and meta-analysis were performed in 2013 and 2015 [9,20], but this was limited to the fluorescence and visual inspection, and for dental carious lesions, we considered the initial caries lesions only. According to International Caries Detection and Assessment System (ICDAS), the initial caries are defined as the demineralized lesion of the enamel surface without a cavity formation, independent of the lesion depth. Thus, the depth of demineralized lesions has not been considered in the study. Another systematic review has been published about non-cavitated carious lesions detection methods, but the authors did not perform meta-analysis [21]. Our systematic review is the first review that has meta-analyses of diagnostic methods of non-cavitated carious lesions. Thus, we have evaluated the accuracy of different methods used to detect non-cavitated caries lesions, the heterogeneity among the studies and the publication bias. Our review intends to emphasize important information for clinicians to choose the appropriate method among fluorescence, visual inspection and visual photographic examination in non-cavitated caries detection.

Visual inspection is the most common method in initial caries detection for its convenience and reliability [9]. However, validating visual inspection in research has several drawbacks. Histology is assumed as the exclusive standard reference leading to difficulties for conducting studies of in vivo visual inspection. To assess the caries in histology, the teeth must be extracted leading to ethical issues. The clinical classification of dental caries is various among studies resulting in the heterogenity of criteria of determining initial caries. Among selected studies, only the study of Teo et al. in 2014 [22], which evaluated the accuracy of in vivo visual inspection, without a meta-analysis, could not generate a relatively good level of evidence.

The ROC curves of in vitro fluorescence and in vitro visual inspection are coincident and are located towards the upper-left corner of the ROC curve of visual photographic examination. The results suggested that in vitro fluorescence and in vitro visual inspection have equivalent accuracy. Studies including a sample of initial caries may use either fluorescence-based methods or visual inspection as research materials. However, the accuracy of in vitro fluorescence and in vivo fluorescence is not equivalent. Thus, using fluorescence in clinical practice instead of visual examination still needs further investigation. Comparing in vivo and in vitro fluorescence, in vitro fluorescence had higher accuracy. Diagnodent pens detect carious lesions through measuring porphyrins released by bacteria and detecting biological luminescence on teeth surfaces. In in vitro studies, teeth were stored inside preservative solutions which may remove and dissolve proteins and wash out microorganisms, then overscore the demineralization level of the lesions [22]. Moreover, in clinical practice, detection of dental caries under restorative materials was impossible with fluorescence-based instruments, and diagnostic performance at the surrounding areas of restorations was also limited [23]. On the other hand, modalities using fluorescence-based methods report more carious lesions than other methods, which means that either the LF pen detects lesions in areas where none really exist, or other devices cannot detect actual lesions. So, the LF pen is not recommended to detect dental caries under restorative materials due to its low performance and should be substituted by optical coherence tomography (OCT) [23]. SOPROLIFE might also give false positive results if images are magnified above a certain threshold [24]. To avoid the false positive result, taking repetitively and comparing between OCT images is a possible solution.

The AUC of in vivo visual photographic examination was lower than that of in vitro visual photographic examination. The result can be explained by the shortcomings of light, photographic direction, humidity, tooth position and saliva control of intraoral environment comparing to laboratory environment [25]. Compared with in vitro visual inspection and in vitro fluorescence, in vitro photographic visual examination had lower AUC. The results can be explained by the effects of photo quality on the distinction of lesions.

The review still has several shortcomings. The number of included studies was small, ten studies of fluorescence, five studies of visual inspection and three studies of photographic visual examination. The QUADAS-2 checklist showed that all articles had a high risk of bias. Another limitation of the review is the heterogeneity of standard references of included studies. Histology was the reference standard of all selected studies that assessed visual inspection, two studies evaluating fluorescence and six studies assessing photographic visual inspection (Appendix C). The reference standard of the other studies evaluating photographic visual inspection and fluorescence was visual inspection, which is a subjective method (Appendix C). Regarding the visual photographic examination, all the included studies were conducted in small samples and used old versions of photographic tools, such as IPhone 5, Nexus 4 and camera Macro [26]. Since all studies included in the study had a high risk of bias, the result of this review should be interpreted with caution.

According to our limited knowledge, the research on non-cavitated caries lesions only has a systematic review in 2013 by Gomez J [21]. Photographic visual examination for diagnosing initial stage caries has been studied by Kohara 2018 [26], Van Hilsen 2013 [27], Seremidi 2012 [28] and has not been reviewed systematically. Fluorescence-based methods and visual inspection have been reviewed with meta-analysis at two different thresholds as enamel and dentine caries lesions. Therefore, this review is the first meta-analysis to compare the accuracy of three diagnostic methods and focus on initial dental caries. Further studies assessing in vivo visual inspection and in vitro and in vivo photographic visual examination are needed with a consistent objective reference standard, i.e., history.

## 5. Conclusions

It is suggested that the visual method and fluorescence method have equivalent accuracy in laboratory use to detect early-stages caries. More studies evaluating in vivo visual inspection and photographic visual examination are required.

## Figures and Tables

**Table 1:** Methodological quality table.

Study	Risk of Bias								Applicability					
	Patient Selection		Index Test		Reference Standard		Flow and Timing		Patient Selection		Index Test		Reference Standard	
	*N*	*%*	*n*	*%*	*N*	*%*	*N*	*%*	*N*	*%*	*N*	*%*	*n*	*%*
High	9	75	6	50	7	58	1	8	0	0	0	0	0	0
Low	3	15	4	33	3	25	11	92	12	100	12	100	12	100
Unclear	0	0	2	17	2	17	0	0	0	0	0	0	0	0
Total	12	100	12	100	12	100	12	100	12	100	12	100	12	100

## Data Availability

Not applicable.

## Appendix A. PubMed Search

PubMed on 30 October 2019.

**Table A1:** PubMed search: diagnostic value of fluorescence methods, visual inspection and photographic visual examination in initial caries lesion.

Search Name	Search Query	Type of Search	Results
#1	Search ((((((sensitive and specificity[MeSH Terms])) OR (sensitivity[Title/Abstract] AND specificity[Title/Abstract] OR sensitivity[Title/Abstract] AND standard[Title/Abstract] OR specificity[Title/Abstract] OR screening[Title/Abstract] OR false positive[Title/Abstract] OR false negative[Title/Abstract] OR accuracy[Title/Abstract])) OR predictive value of tests[MeSH Terms]) OR (predictive value[Title/Abstract] OR predictive value of tests[Title/Abstract] OR predictive value of standard[Title/Abstract] OR predictive values[Title/Abstract] OR reference value[Title/Abstract] OR reference values[Title/Abstract] OR reference values[Title/Abstract] OR reference standards[Title/Abstract])) OR roc curve[MeSH Terms]) OR (roc[Title/Abstract] OR roc analyses[Title/Abstract] OR roc analysis[Title/Abstract] OR roc area[Title/Abstract] OR roc auc[Title/Abstract] OR roc characteristics[Title/Abstract] OR roc curve method[Title/Abstract] OR roc estimated[Title/Abstract] OR roc evaluation[Title/Abstract] OR likelihood ratio[Title/Abstract])	MeSH terms Title/Abstract	1,529,366
#2	Search ((initial caries OR white spots[MeSH Terms])) OR (caries in early phase[Title/Abstract] OR initial phase of dental caries[Title/Abstract] OR first stage of tooth decay[Title/Abstract] OR White Spots[Title/Abstract] OR first stage of cavities[Title/Abstract] OR decay on the surface of the teeth[Title/Abstract] OR early stages caries[Title/Abstract] OR early stages decay[Title/Abstract] OR Early stage of carious lesion[Title/Abstract] OR early tooth decay[Title/Abstract] OR Early-stage tooth decay[Title/Abstract] OR initial phase of tooth decay[Title/Abstract])	MeSH terms Title/Abstract	46,540
#3	Search (((system[Title/Abstract] OR clinical[Title/Abstract] OR clinic[Title/Abstract] OR exams[Title/Abstract] OR examination[Title/Abstract] OR examinations[Title/Abstract] OR visual[Title/Abstract] OR inspection[Title/Abstract])) OR (laser fluorescence[Title/Abstract] OR DIAGNOdent[Title/Abstract] OR infrared[Title/Abstract] OR diode laser fluorescence[Title/Abstract] OR QLF[Title/Abstract] OR quantitative light-induced fluorescence system[Title/Abstract] OR quantitative light-induced fluorescence[Title/Abstract] OR fluorescence-bases methods[Title/Abstract] OR fluorescence camera[Title/Abstract] OR VistaProof-FC[Title/Abstract] OR VistaProof[Title/Abstract])) OR (“photographic[Title/Abstract] OR smartphone based method[Title/Abstract] OR photography[Title/Abstract] OR smartphone images[Title/Abstract] OR smartphone photograph[Title/Abstract] OR oral photographic [Title/Abstract] OR smartphone-based detection[Title/Abstract] OR smartphone-based diagnostics[Title/Abstract] OR image-based detection[Title/Abstract] OR smartphone-based tool[Title/Abstract] OR smartphone-based screening”[Title/Abstract])	Title/Abstract	8,967,806
#4	#1 AND #2 AND #3		684

## Appendix B. Cochrane Search

Cochrane on 30 October 2019.

**Table A2:** Cochrane search: diagnostic value of fluorescence methods, visual inspection and photographic visual examination in initial caries lesion.

Search Name	Search Query	Type of Search	Results
#1	Search ((((((sensitive and specificity[MeSH Terms])) OR (sensitivity[Title/Abstract] AND specificity[Title/Abstract] OR sensitivity[Title/Abstract] AND standard[Title/Abstract] OR specificity[Title/Abstract] OR screening[Title/Abstract] OR false positive[Title/Abstract] OR false negative[Title/Abstract] OR accuracy[Title/Abstract])) OR predictive value of tests[MeSH Terms]) OR (predictive value[Title/Abstract] OR predictive value of tests[Title/Abstract] OR predictive value of standard[Title/Abstract] OR predictive values[Title/Abstract] OR reference value[Title/Abstract] OR reference values[Title/Abstract] OR reference values[Title/Abstract] OR reference standards[Title/Abstract])) OR roc curve[MeSH Terms]) OR (roc[Title/Abstract] OR roc analyses[Title/Abstract] OR roc analysis[Title/Abstract] OR roc area[Title/Abstract] OR roc auc[Title/Abstract] OR roc characteristics[Title/Abstract] OR roc curve method[Title/Abstract] OR roc estimated[Title/Abstract] OR roc evaluation[Title/Abstract] OR likelihood ratio[Title/Abstract])	MeSH terms Title/Abstract	115,527
#2	Search ((initial caries OR white spots[MeSH Terms])) OR (caries in early phase[Title/Abstract] OR initial phase of dental caries[Title/Abstract] OR first stage of tooth decay[Title/Abstract] OR White Spots[Title/Abstract] OR first stage of cavities[Title/Abstract] OR decay on the surface of the teeth[Title/Abstract] OR early stages caries[Title/Abstract] OR early stages decay[Title/Abstract] OR Early stage of carious lesion[Title/Abstract] OR early tooth decay[Title/Abstract] OR Early-stage tooth decay[Title/Abstract] OR initial phase of tooth decay[Title/Abstract])	MeSH terms Title/Abstract	2480
#3	Search (((system[Title/Abstract] OR clinical[Title/Abstract] OR clinic[Title/Abstract] OR exams[Title/Abstract] OR examination[Title/Abstract] OR examinations[Title/Abstract] OR visual[Title/Abstract] OR inspection[Title/Abstract])) OR (laser fluorescence[Title/Abstract] OR DIAGNOdent[Title/Abstract] OR infrared[Title/Abstract] OR diode laser fluorescence[Title/Abstract] OR QLF[Title/Abstract] OR quantitative light-induced fluorescence system[Title/Abstract] OR quantitative light-induced fluorescence[Title/Abstract] OR fluorescence-bases methods[Title/Abstract] OR fluorescence camera[Title/Abstract] OR VistaProof-FC[Title/Abstract] OR VistaProof[Title/Abstract])) OR (“photographic[Title/Abstract] OR smartphone based method[Title/Abstract] OR photography[Title/Abstract] OR smartphone images[Title/Abstract] OR smartphone photograph[Title/Abstract] OR oral photographic [Title/Abstract] OR smartphone-based detection[Title/Abstract] OR smartphone-based diagnostics[Title/Abstract] OR image-based detection[Title/Abstract] OR smartphone-based tool[Title/Abstract] OR smartphone-based screening”[Title/Abstract])	Title/Abstract	854,978
#4	#1 AND #2 AND #3		113

## Appendix C. Summary of Characteristics of Included Studies

**Table A3:** Summary of characteristics of included studies.

Fluorescence Method												
Study ID	Database	N	TP	FP	FN	TN	Method	Primary/Permanent	Tooth Surface	In Vitro/In Vivo	Reference Standard	Cut-Off Value
Iranzo-Cortes et al. 2018 [1]	PubMed	65	31	4	13	17	FC	permanent	smooth	in vitro without frozen	histology	sound (1–1.49); initial caries (1.5–1.99); caries enamel (2–2.49); caries dentine (2.5 or higher)
Mansour et al. 2016 [3]	PubMed	426	30	15	21	360	LF pen	permanent	coronal	in vivo	visual and radiography	sound (0–13); outer half enamel (14–20); internal half enamel (21–29); dentinal (>30)
Ozsevik et al. 2015 [4]	Cochrane	156	92	23	7	34	LFpen	permanent	proximal caries	in vitro frozen	histology	sound (0–9); outer half enamel (9.1–15); internal half enamel (>15)
Zeitouny et al. 2014 [5]	PubMed	164	104	6	8	46	FC	permanent	occlusal	in vivo	visual	sound (shiny green); enamel (red–darker red); dentinal (dark red–red orange)
Teo et al. 2014a [6]	PubMed	102	67	2	11	22	LF pen	primary	occlusal	in vivo	histology	optimal cut off D1:10
Teo et al. 2014a1 [6]	PubMed	64	40	10	6	8	LF pen	primary	occlusal	in vitro without frozen	histology	optimal cut off D1:10
Achilleos et al. 2013a [7]	PubMed	38	27	2	9	0	LF pen	permanent	occlusal	in vitro without frozen	histology	sound (0–13); outer half enamel (14–20); internal half enamel (21–29); dentinal (>30)
Achilleos et al. 2013a1 [7]	PubMed	38	36	2	0	0	FC	permanent	occlusal	in vitro without frozen	histology	sound (≤ 1); beginning enamel (1–1.5); deep enamel (2–2.5); dentinal (2.5–5)
Seremidi et al. 2012a [9]	PubMed	107	66	6	17	18	LF pen	permanent	occlusal	in vitro without frozen	histology	sound (<9); enamel (9–44); dentinal (>=44)
Seremidi et al. 2012a1 [9]	PubMed	107	71	7	12	17	FC	permanent	occlusal	in vitro without frozen	histology	sound (<1.3); D1 (1.30); D2 (1.41); D3 (>1.59)
Duruturk et al. 2011 [10]	PubMed	505	163	105	20	217	LF	permanent	occlusal	in vivo	visual	Sound (0–14); enamel (15–20); dentinal (≥21)
Matos et al. 2011a [11]	PubMed	383	241	6	110	26	LFpen	primary	occlusal	in vivo	visual inspection	sound (0–4); NC lesions (>4)
Matos et al. 2011a1 [11]	PubMed	383	156	3	195	29	FC	primary	occlusal	in vivo	visual inspection	sound (0–1.1); NC lesions (>1.1)
de Paula et al. 2011a [12]	PubMed	64	40	0	16	8	LF	permanent	occlusal	in vitro without frozen	histology	sound (0–10); enamel (11–20); dentin (21–99)
de Paula et al. 2011a1 [12]	PubMed	64	31	0	25	8	LF	permanent	occlusal	insitu without frozen	histology	sound (0–10); enamel (11–20); dentin (21–99)
**Visual Inspection**												
**Study ID**	**Database**	**N**	**TP**	**FP**	**FN**	**TN**	**Criteria**	**Primary/Permanent**	**Tooth Surface**	**In Vitro/In Vivo**	**Reference Standard**	**Examiners’ Experience**
Iranzo-Cortes et al. 2018 [1]	PubMed	65	35	4	9	17	ICDAS II	permanent	smooth	in vitro without frozen	histology	novices with training
Teo et al. 2014a [6]	PubMed	102	71	7	7	17	ICDAS	primary	occlusal	in vivo	histology	intermediate with training
Teo, et al. 2014a1 [6]	PubMed	64	41	7	5	11	ICDAS	primary	occlusal	in vitro without frozen	histology	intermediate with training
Achilleos, et al. 2013 [7]	PubMed	38	29	2	7	0	ICDAS	permanent	occlusal	in vitro without frozen	histology	experienced
Seremidi et al. 2012a [9]	PubMed	107	73	11	10	13	Ekstrand	permanent	occlusal	in vitro without frozen	histology	experienced
de Paula, et al. 2011a [12]	PubMed	64	35	0	21	8		permanent	occlusal	in vitro without frozen	histology	intermediate without training
de Paula, et al. 2011a1 [12]	PubMed	64	30	1	26	7		permanent	occlusal	in situ	histology	intermediate without training
